# Impaired renal function and dysbiosis of gut microbiota contribute to increased trimethylamine-N-oxide in chronic kidney disease patients

**DOI:** 10.1038/s41598-017-01387-y

**Published:** 2017-05-03

**Authors:** Kai-Yu Xu, Geng-Hong Xia, Jun-Qi Lu, Mu-Xuan Chen, Xin Zhen, Shan Wang, Chao You, Jing Nie, Hong-Wei Zhou, Jia Yin

**Affiliations:** 1 Department of Neurology, NanFang Hospital, Southern Medical University, Guangzhou, China; 2 Department of Environmental Health, School of Public Health, Southern Medical University, Guangzhou, China; 3 State Key Laboratory of Organ Failure Research, Division of Laboratory Medicine, ZhuJiang Hospital, Southern Medical University, Guangzhou, China; 4 Department of Nephrology, NanFang Hospital, Southern Medical University, Guangzhou, China

## Abstract

Chronic kidney disease (CKD) patients have an increased risk of cardiovascular diseases (CVDs). The present study aimed to investigate the gut microbiota and blood trimethylamine-N-oxide concentration (TMAO) in Chinese CKD patients and explore the underlying explanations through the animal experiment. The median plasma TMAO level was 30.33 μmol/L in the CKD patients, which was significantly higher than the 2.08 μmol/L concentration measured in the healthy controls. Next-generation sequence revealed obvious dysbiosis of the gut microbiome in CKD patients, with reduced bacterial diversity and biased community constitutions. CKD patients had higher percentages of opportunistic pathogens from gamma-Proteobacteria and reduced percentages of beneficial microbes, such as *Roseburia*, *Coprococcus*, and Ruminococcaceae. The PICRUSt analysis demonstrated that eight genes involved in choline, betaine, L-carnitine and trimethylamine (TMA) metabolism were changed in the CKD patients. Moreover, we transferred faecal samples from CKD patients and healthy controls into antibiotic-treated C57BL/6 mice and found that the mice that received gut microbes from the CKD patients had significantly higher plasma TMAO levels and different composition of gut microbiota than did the comparative mouse group. Our present study demonstrated that CKD patients had increased plasma TMAO levels due to contributions from both impaired renal functions and dysbiosis of the gut microbiota.

## Introduction

Chronic kidney disease (CKD) is characterized by impaired kidney functions or increased proteinuria confirmed on two or more occasions at least 3 months apart^1^. CKD has become an important public health problem in China, with an overall prevalence of 10.8% (~119.5 million people)^2^. CKD is considered an independent cardiovascular risk factor, and a CKD diagnosis implies a very high cardiovascular risk. Thus, cardiovascular disease (CVD) is one of the major comorbidities of CKD. Some studies have revealed a direct correlation between the CVD prevalence and CKD severity^3^. A Chinese epidemiological study revealed that CVD accounted for nearly 44.2–51.0% of the overall mortality among CKD dialysis patients, which represented a 50-fold increase over the prevalence in the general population^4^. The complex association of CKD with CVD is probably due to several cardiovascular risk factors, including traditional cardiovascular risk factors, such as hypertension, diabetes mellitus, and dyslipidemia^5^, and many novel risk factors, such as anaemia, proteinuria, systemic inflammation, and oxidative stress^6^.

Recently, the gut microbiota has been found to play critical roles in a variety of diet-related chronic inflammatory diseases, including obesity, type 2 diabetes, insulin resistance, atherosclerosis and nonalcoholic fatty liver disease^7–9^. Dysbiosis of the gut microbial community in the development of CKD has attracted attention. Vaziri *et al*. found that ESRD patients had higher gut bacterial diversity than the control group^10^ and reported an increase in microbes from the phyla Firmicutes, Actinobacteria and Proteobacteria in ESRD patients and a decrease in Bifidobacteria and Lactobacilli. Additionally, a recent study demonstrated that intestinal microbiome dysbiosis led to intestinal barrier dysfunction and bacterial translocation, which finally triggered a state of persistent systemic inflammation in CKD patients^11^. Within the gut microbiota-derived metabolites, trimethylamine-N-oxide (TMAO) is one of the most important novel risk factors for CVD and related diseases^12^. Ingested nutrients, such as phosphatidylcholine and L-carnitine, can be transformed by gut microbes into trimethylamine, which is absorbed and oxidized by hepatic flavin monooxygenase (FMO) to TMAO and finally excreted by the kidneys. A 5-year follow up study demonstrated that circulating TMAO was a forceful prognostic marker for adverse cardiac events^13^. Moreover, elevated TMAO concentrations are positively correlated with the long-term mortality risk in patients with atherosclerosis, heart failure, and CKD^14, 15^. Kim *et al*. reported that the TMAO levels were significantly elevated in CKD patients^16^, indicating that TMAO also played a role as a risk factor in these patients. Thus, we could assume that the elevated TMAO levels are caused by the dysbiosis of gut microbiota and impaired renal functions in Chinese CKD patients.

Recently, we reported that the TMAO level in Chinese atherosclerosis patients was similar to the level observed in the control group but was deceased in stroke patients, which was different from data reported from studies in the USA^17, 18^. This phenomenon suggested that TMAO might be affected by different dietary habitats and gut microbial communities in different regions. In the present study, we evaluated the gut microbial community and the TMAO levels in Chinese CKD patients and explored the underlying explanations through the animal experiment.

## Results

### CKD patients showed worse clinical manifestations and significantly elevated TMAO concentrations, compared to the healthy participants

To investigate the clinical conditions and plasma TMAO concentrations of the CKD patients, we collected basic clinical indices and measured TMAO concentrations for analysis. The CKD patients and control individuals enrolled in the present study were comparable in age and gender (Table 1). The CKD patients had a relatively higher burden of comorbidities compared with the healthy individuals, including diabetes mellitus, CVD and metabolic syndrome. Additionally, the CKD patients showed obviously impaired renal functions and anaemia symptoms, including significantly elevated blood urea nitrogen and creatinine and reduced red blood cells and haemoglobin. Symptoms related to higher cardiovascular system burdens, including significantly elevated systolic blood pressure and diastolic blood pressure and a reduced plasma high density lipoprotein level, were also observed in the CKD group. Next, we divided the CKD patients into two subgroups according to their glomerular filtration rates (GFRs). The high GFR group had 16 members with GFR values higher than or equal to 7 ml/min/1.73 m^2^, which represented a relatively low level of renal impairment, whereas the low GFR group (GFR < 7 ml/min/1.73 m^2^) also had 16 individuals. As demonstrated in Table 2, the low GFR group patients had relatively higher levels of L-carnitine and cystatin-C compared to the high GFR group patients.

**Table 1:** Characteristics of the study participants.

	Patients (n = 32)	Controls (n = 32)	P-value
Numbers	32	32	Not available
Age	53.34 (14.47)	55.03 (10.38)	0.717
Gender, M(%)	16 (50)	16 (50)	Not available
WBC, 109/L^#^	7.18 (3.03)	6.37 (2.39)	0.087
RBC, 1012/L^#^	3.05 (0.86)	4.76 (0.62)	<0.001***
HGB, g/L	88.81 (19.47)	141.54 (15.07)	<0.001***
PLT, 109/L	211.21 (86.64)	242.67 (49.65)	0.068
GLU, mmol/L^#^	4.66 (1.49)	4.80 (0.78)	0.821
BUN, mmol/L^#^	16.74 (8.41)	4.53 (1.57)	<0.001***
Cr, µmol/L^#^	522.50 (339.50)	66.00 (20.75)	<0.001***
SBP, mmHg^#^	141.00 (42)	120.50 (23.25)	<0.001***
DBP, mmHg	79.30 (15.35)	72.06 (10.76)	0.040*
TC, mmol/L	4.69 (1.23)	5.10 (0.76)	0.078
LDL, mmol/L^#^	2.74 (1.15)	3.26 (1.05)	0.053
HDL, mmol/L	1.08 (0.28)	1.21 (0.20)	0.038*
VLDL, mmol/L^#^	0.55 (0.57)	0.66 (0.36)	0.379
TG, mmol/L^#^	1.35 (1.00)	1.25 (1.06)	0.201
Comorbidities, n (%)			
Diabetes	12 (38)	0 (0)	Not available
Metabolic syndrome	23 (72)	0 (0)	Not available
CVD	25 (78)	0 (0)	Not available
Cause of kidney disease, n (%)			
Polycystic kidney disease	1 (3)		
Nephrosclerosis	10 (31)		
Diabetic nephropathy	5 (15)		
Glomerulonephritis	4 (13)		
Unknown or other aetiology	12 (38)		

Data represent the mean (standard deviation), and the data ^#^represent the median (interquartile range). For all records, the P values represent the results of the Mann-Whitney U test. WBC, white blood cell count; RBC, red blood cell; HGB, haemoglobin; PLT, platelet; GLU, glucose; BUN, blood urea nitrogen; Cr, creatinine; SBP, systolic blood pressure; DBP, diastolic blood pressure; TC, total cholesterol; LDL, low-density lipoprotein; HDL, high-density lipoprotein; VLDL, very low-density lipoprotein; TG, triglycerides; CVD, cardiovascular disease. *P < 0.05; **P < 0.01; ***P < 0.001.

**Table 2:** Characteristics of the high GFR group and the low GFR group.

	High GFR group (n = 16)	Low GFR group (n = 16)	P-value
Numbers	16	16	Not available
Age	55.43 (11.42)	51.25 (17.12)	0.509
WBC, 109/L	7.28 (2.44)	8.19 (2.11)	0.152
RBC, 1012/L	3.22 (0.63)	2.90 (0.63)	0.132
HGB, g/L	94.06 (16.44)	83.56 (21.32)	0.122
PLT, 109/L	227.31 (71.95)	195.12 (98.92)	0.181
GLU, mmol/L^#^	4.61 (2.13)	4.99 (1.25)	0.979
BUN, mmol/L	16.69 (5.95)	16.55 (6.64)	0.880
Cr, µmol/L	548.50 (256.16)	650.62 (294.34)	0.346
CRP, mg/L^#^	1.75 (18.93)	10.34 (19.85)	0.150
Cystatin-C, mg/L	4.93 (1.51)	6.60 (2.17)	0.013*
SBP, mmHg	150.64 (19.42)	137.37 (29.99)	0.157
DBP, mmHg^#^	83.50 (16.00)	83.00 (30.25)	0.148
TC, mmol/L	4.28 (0.99)	5.07 (1.35)	0.089
LDL, mmol/L^#^	2.35 (0.90)	2.88 (1.70)	0.089
HDL, mmol/L	1.10 (0.33)	1.05 (0.24)	0.890
VLDL, mmol/L^#^	0.54 (0.24)	0.58 (0.74)	0.489
TG, mmol/L^#^	1.24 (0.60)	1.60 (1.05)	0.133
TMA, μmol/L^#^	8.71 (1.81)	9.83 (1.93)	0.051
L-carnitine, μmol/L^#^	49.39 (24.91)	131.13 (195.79)	0.036*

Data represent the mean (standard deviation), and the data ^#^represent the median (interquartile range). For all records, the P values represent the results of the Mann-Whitney U test. WBC, white blood cell count; RBC, red blood cell; HGB, haemoglobin; PLT, platelet; GLU, glucose; BUN, blood urea nitrogen; Cr, creatinine; CRP, c-reactive protein; SBP, systolic blood pressure; DBP, diastolic blood pressure; TC, total cholesterol; LDL, low-density lipoprotein; HDL, high-density lipoprotein; VLDL, very low-density lipoprotein; TG, triglycerides; TMA, trimethylamine. *P < 0.05; **P < 0.01; ***P < 0.001.

**Figure 1:**
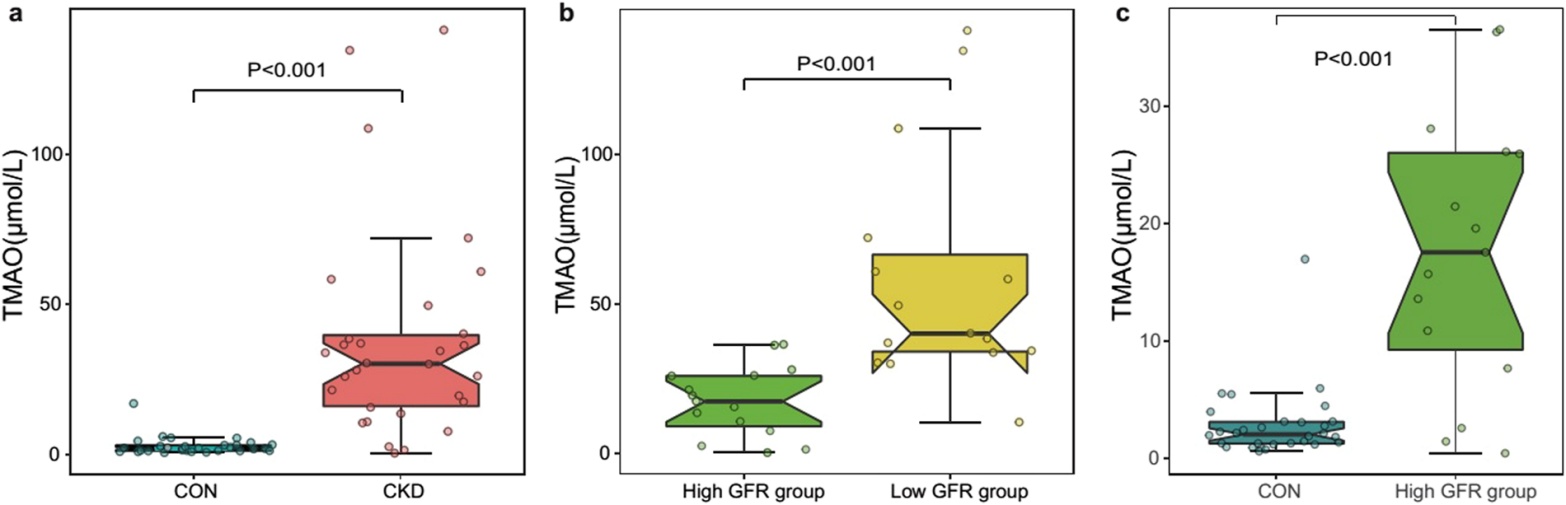
CKD patients showed significantly elevated plasma TMAO concentrations. (**a**) Comparison of plasma TMAO levels between the CKD patient group and the control group. (**b**) Comparison of plasma TMAO levels between the high GFR group (GFR ≥ 7 ml/min/1.73 m^2^) and the low GFR group (GFR < 7 ml/min/1.73 m^2^). (**c**) Comparison of plasma TMAO levels between the high GFR group (GFR ≥ 7 ml/min/1.73 m^2^) and the control group. TMAO, trimethylamine N-oxide; CKD, chronic kidney disease; GFR, glomerular filtration rate. The results are based on a Mann-Whitney U test of the TMAO concentrations.

The TMAO concentration was determined using LC-MS/MS with a stable isotope as the internal standard. The CKD patients had obviously higher TMAO concentrations than the healthy controls (Fig. 1a). The fasting blood TMAO concentration was 30.33 ± 27.35 μmol/L in the CKD patients and only 2.08 ± 1.89 μmol/L in the healthy controls (Mann-Whitney U test, P < 0.001). The TMAO concentrations between the high GFR group and the low GFR group were significantly different (17.58 ± 18.45 μmol/L and 40.23 ± 38.10 μmol/L, respectively) (Fig. 1b) (Mann-Whitney U test, P < 0.001). Compared to the control group, the high GFR group showed significantly elevated TMAO concentrations (Fig. 1c) (Mann-Whitney U test, P < 0.001).

In conclusion, compared to the healthy controls, CKD patients showed worse clinical manifestations and significantly elevated TMAO concentrations. The low GFR group showed significantly higher TMAO concentrations than the high GFR group, which showed significantly higher TMAO concentrations than the control group.

### Obvious dysbiosis of the gut microbiota in CKD patients, including reduced bacterial α–diversity and biased community constitutions

**Figure 2:**
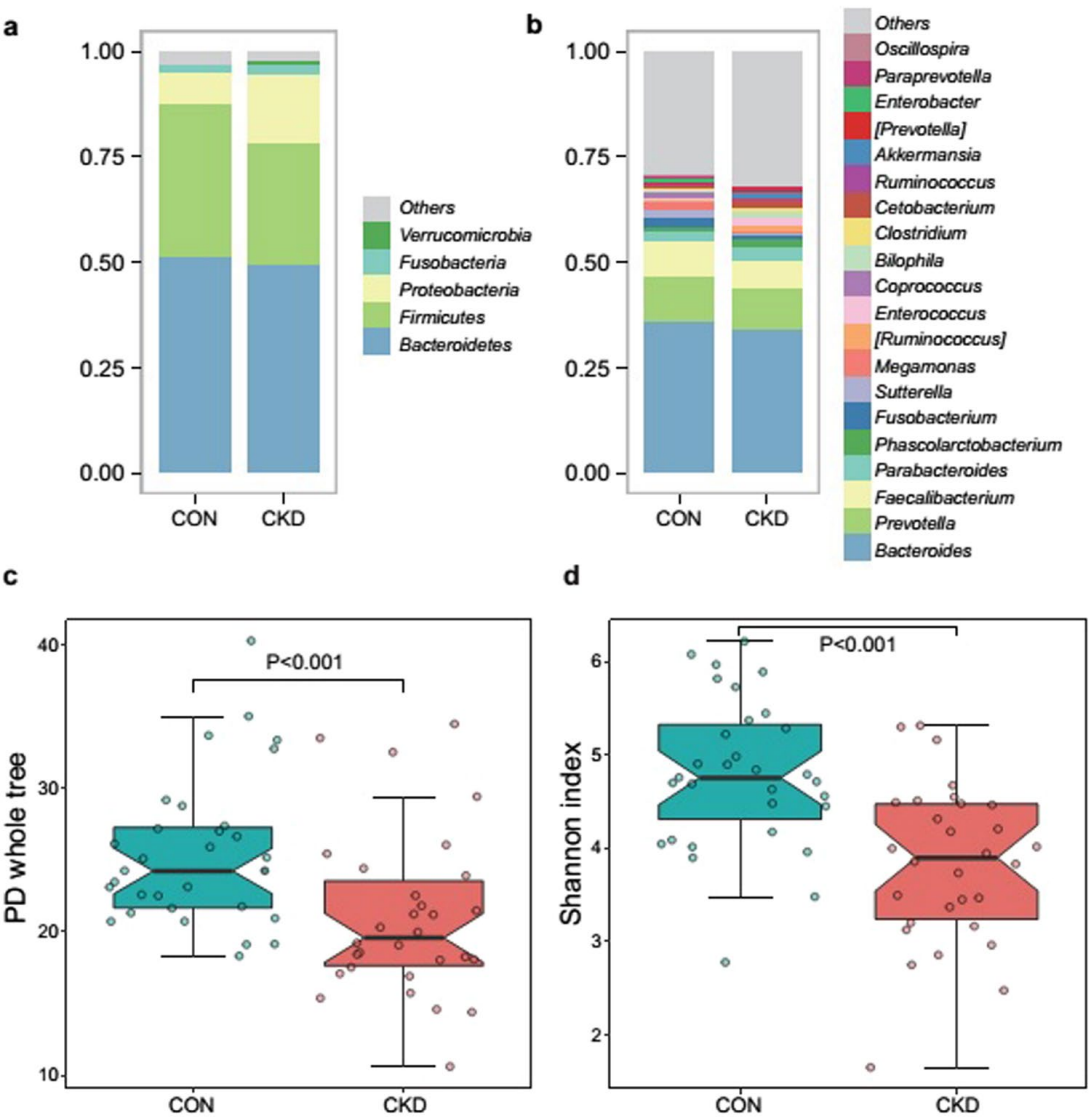
The healthy controls exhibited significantly greater bacterial diversity than the patients. The data represent the comparison of gut bacterial profiles between the healthy controls and CKD patients, including 31 healthy controls (blue) and 30 CKD patients (red). (**a,b**) Average relative abundances of the predominant bacterial taxa at the phylum level and the genus level in the CKD patient and control samples. (**c,d**) Comparison of α-diversity between the gut microbiota of the CKD patients and controls. We used two indices to represent the α-diversity (the Shannon index and the PD whole tree). PD indicates phylogenetic diversity. The Wilcoxon rank sum test was used to determine significance in α-diversity.

To investigate the microbial composition in study participants, we conducted the basic analysis in this area. Both the healthy controls and the CKD patients had typical human microbiome structures. Most bacteria fell into the phyla Bacteroidetes, Firmicutes, Proteobacteria, Fusobacteria, and Verrucomicrobia. At the genus level, *Bacteroides*, *Prevotella*, *Faecalibacterium*, *Parabacteroides*, and *Phascolarctobacterium* occupied the highest abundance. Although these bacterial genera were detected in both groups, some of the genera showed differences between the groups (Fig. 2a,b).

The α–diversity indices are commonly used to describe the ecological diversity within microbial community samples. The phylogenetic diversity whole tree index (PD-whole tree) represents the phylogenetic diversity of the whole microbial community, whereas the Shannon index considers both the species richness and evenness. According to the Wilcoxon rank sum test analysis, both of these indices were significantly lower in the CKD group (P < 0.001) (Fig. 2c,d).

**Figure 3:**
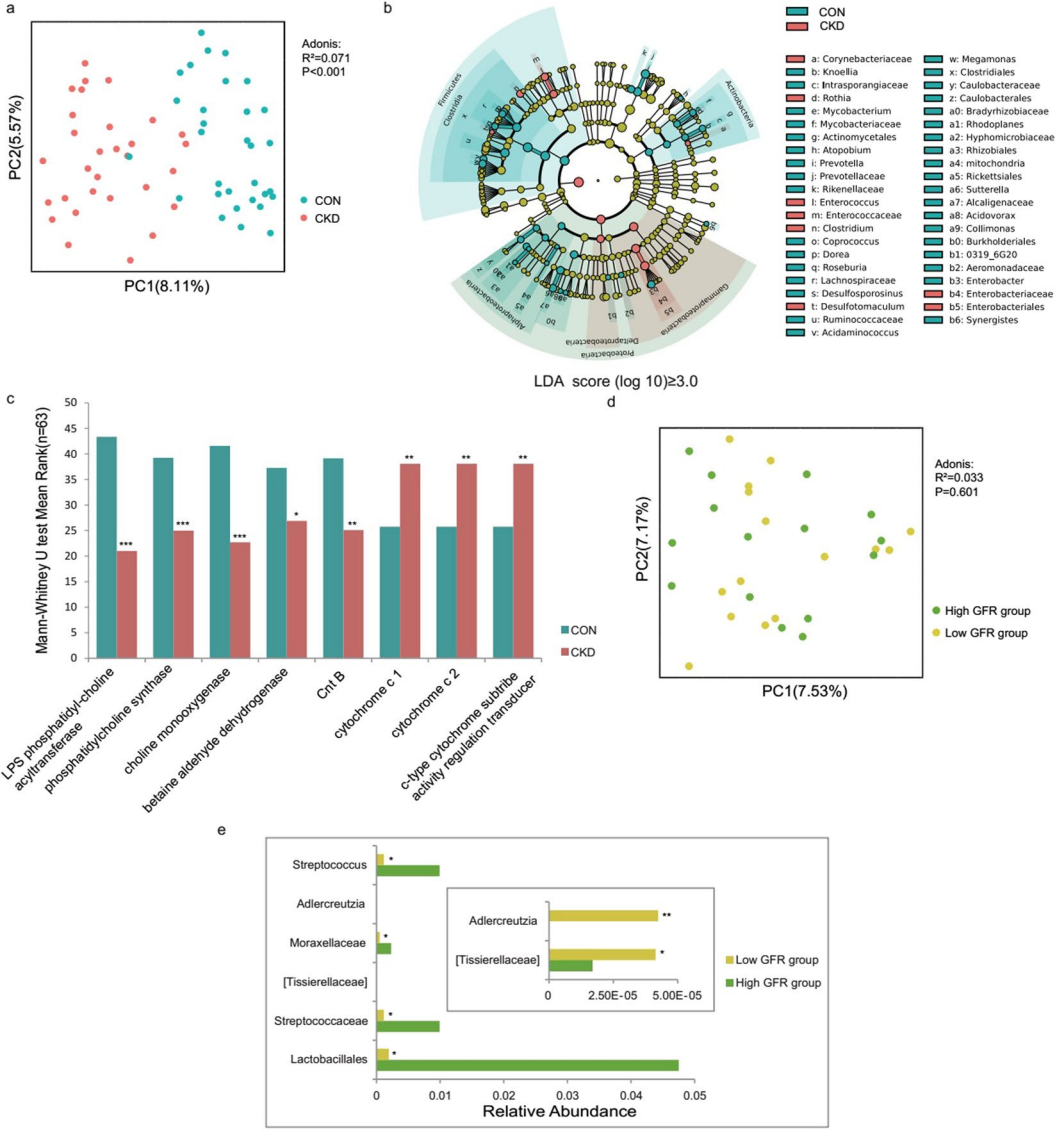
CKD patients showed obvious dysbiosis of the gut microbiome. The data represent the comparison of the gut bacterial profiles between the CKD patients and the healthy controls, including 31 healthy controls (blue) and 30 CKD patients (red). (**a**) Principal coordinate analysis illustrating the grouping patterns of the CKD patient group and the control group based on the unweighted UniFrac distances. Each closed circle represents a sample. Distances between any pair of samples represent their dissimilarities. (**b**) Significantly discriminative taxa between the patients and controls were determined using Linear Discriminant Analysis Effect Size (LEfSe). Only taxa meeting the LDA significance thresholds (>3) are shown. Different coloured regions represent different groups. From the interior to the exterior, each layer represents the phylum, class, order, family, and genus level. (**c**) Prediction of gene functions between the CKD patients and the controls. Different coloured bar charts represent different groups. (**d**) Comparison of gut bacterial profiles between two CKD patient subgroups: the high GFR group (GFR ≥ 7 ml/min/1.73 m^2^) and the low GFR group (GFR < 7 ml/min/1.73 m^2^). Principal coordinate analysis illustrating the grouping patterns of the CKD patients based on the unweighted UniFrac distances. The data represent 15 GFR high patients (green) and 15 GFR low patients (yellow). Each closed circle represents a sample. (**e**) Comparison of raw gut mcirobiome data between two CKD patient subgroups. Different coloured bar charts represent different groups. The Mann-Whitney U test was used to determine significance between groups. *P < 0.05; **P < 0.01; ***P < 0.001.

PCoA is a dimensionality reduction method that illustrates the relationship between samples depending on the distance matrix and visualizes the unsupervised grouping pattern of a complex data set, such as the microbiome. Here, we used UniFrac distances to describe the phylogenetic dissimilarity among samples. A smaller UniFrac distance between two samples indicates a higher similarity among the two microbial communities^19^. According to the unweighted UniFrac distance analysis, the distance between the CKD group and the control group was significantly different (ADONIS analysis, P < 0.001, R^2^ = 0.071), indicating that the bacterial composition was significantly different between the groups (Fig. 3a).

In conclusion, next-generation sequence profiling of the gut microbiota revealed obvious dysbiosis of the gut microbiota in CKD patients, including reduced bacterial α–diversity and biased community constitutions.

### CKD patients showed different dominant bacteria and altered intestinal genes expression, compared to the control group

To identify taxonomic differences between the CKD patients and healthy controls, we used the LEfSe tool with a relatively stringent LDA value of 3 (Fig. 3b). At the phylum level, the CKD patients showed increased Proteobacteria but reduced Firmicutes and Actinobacteria. At the family level, the CKD patients showed increased Enterobacteriaceae and Corynebacteriaceae but reduced Ruminococcaceae. At the genus level, *Enterococcus* and *Clostridium* were enriched in the CKD patients, whereas *Prevotella, Coprococcus, Megamonas, Sutterella, Enterobacter, Acidaminococcus, Dorea*, and *Roseburia* were more abundant in the healthy individuals.

To deduce whether these microbial community differences were related to TMAO production, we performed a PICRUSt analysis to infer the functional profiles of the microbiome communities^20^. We found 6910 genes in total, among which 30 genes correlated with choline, betaine, carnitine and trimethylamine metabolism. Within these genes, 8 genes differed significantly between the CKD patients and the healthy controls (Mann-Whitney U test, P < 0.05) (Fig. 3c). Genes related to choline, betaine and L-carnitine metabolism were significantly depleted in the CKD patients, including K07271 (functional annotation: LPS phosphatidyl-choline acyltransferase), K01004 (functional annotation: phosphatidylcholine synthase), K00499 (functional annotation: choline monooxygenase), K00130 (functional annotation: betaine aldehyde dehydrogenase), and K00540 (functional annotation: Cnt B). The significantly increased genes in the CKD patients were related to TMA production in the intestinal tract, including K07811 (functional annotation: trimethylamine oxidoreductase (cytochrome c) 1), K07821 (functional annotation: trimethylamine oxidoreductase (cytochrome c) 2, c-type cytochrome subtribe activity regulation transducer), and K03532 (functional annotation: trimethylamine oxidoreductase (cytochrome c) 1, c-type cytochrome subtribe activity regulation transducer).

In summary, CKD patients showed different dominant bacteria and altered genes expression, which involved in choline, betaine, L-carnitine and trimethylamine (TMA) metabolism, compared to the control group.

### There were no significant differences of microbial composition between the low GFR group and the high GFR group

Due to the different TMAO levels between the GFR groups, we attempted to evaluate differences in their bacterial communities. No obvious difference was observed in the PCoA (ADONIS analysis, P = 0.601, R^2^ = 0.033) (Fig. 3d). By analyzing the raw microbiome data of CKD patients (Fig. S1), we further found that the low GFR group harbored reduced *Streptococcus*, Streptococcaceae, and Lactobacillales, compared to the high GFR group (Fig. 3e). Furthermore, the low GFR group showed increased *Adlercreutzia*, which was positively correlated to the TMAO level (Spearman analysis, P = 0.002, R = 0.528), but the abundance of *Adlercreutzia* was relatively low (<0.00005). These results indicated that the difference of microbial composition between the low GFR group and the high GFR group was not significant.

### Transplantation of the CKD patient microbiota induced an increased TMAO level and dysbiosis of the gut microbiota in antibiotic-treated mice

**Figure 4:**
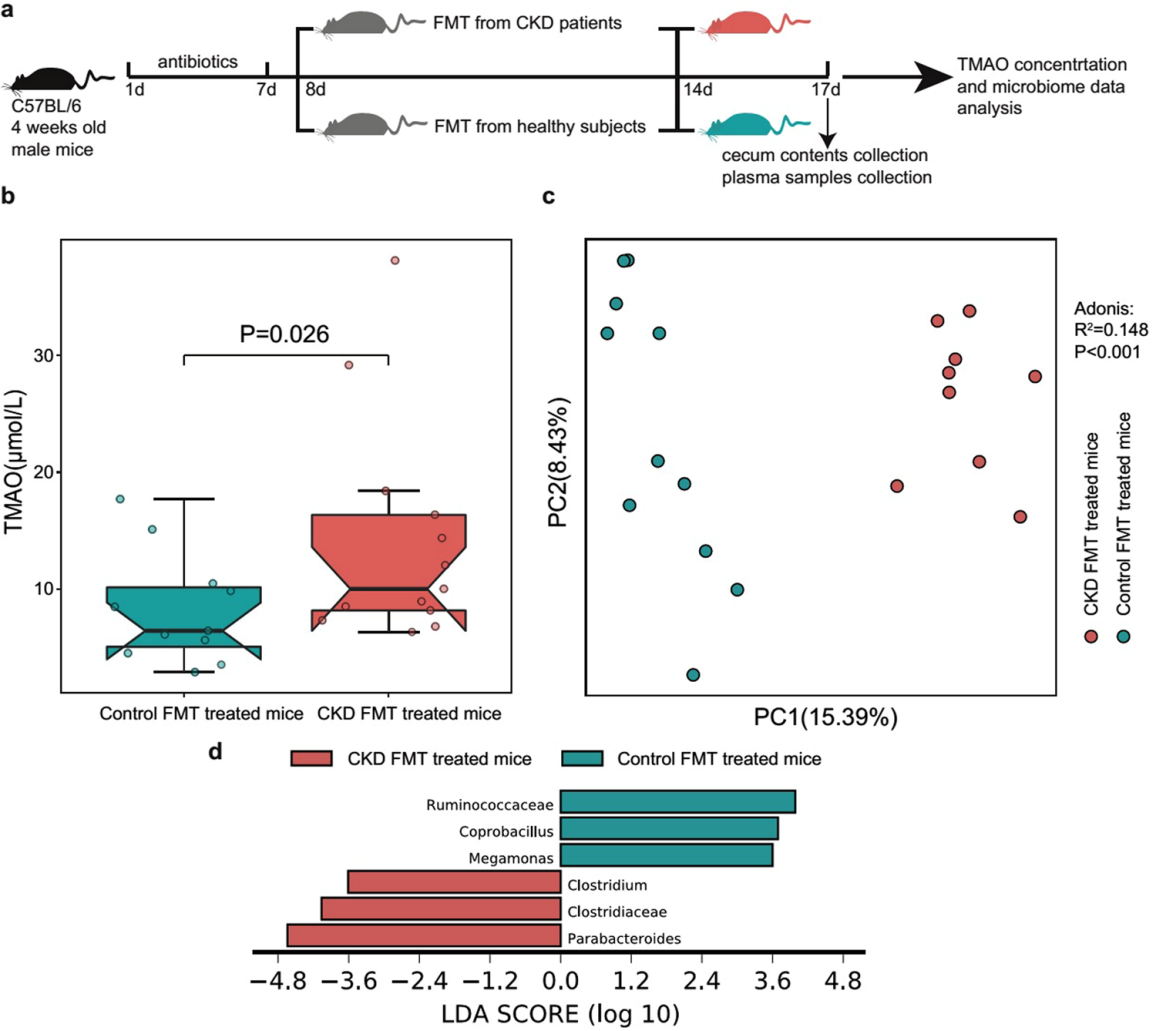
Transplantation of the CKD patient microbiota induces an increased TMAO level and dysbiosis of the gut microbiota in antibiotic-treated mice. The data represent 12 mice transplanted with healthy control faecal samples (blue) and 13 mice transplanted with CKD patient faecal samples (red). (**a**) Experimental design of the faecal microbiota transplantation (FMT) in antibiotic-treated mice. (**b**) Comparison of the plasma TMAO levels between the groups of mice after transplant with pooled faecal samples from the CKD patients and healthy controls. The Mann-Whitney U test was used to determine significance between groups. (**c**) Principal coordinate analysis illustrating the grouping patterns of the two groups of mice based on the unweighted UniFrac distances. Each open circle represents a sample. Distances between any pair of samples represent their dissimilarities. (**d**) Significantly discriminative taxa between the two group of mice were determined using Linear Discriminant Analysis Effect Size (LEfSe). Only taxa meeting the LDA significance thresholds (>3) are shown. Different coloured bars represent different groups.

To investigate whether changes in the gut microbiome contributed to the increased TMAO level in the CKD patients, we subjected antibiotic-treated mice to the FMT technique (Fig. 4a)^21^. The mouse group that received the gut microbiota from the CKD patients showed a significantly higher TMAO concentration compared to the group that received faecal microbes from the healthy controls (Mann-Whitney U test, P = 0.026) (Fig. 4b). The composition of the gut microbiota in mice that received the faecal samples from CKD patients was significantly different from that from the control (ADONIS analysis, P < 0.001, R^2^ =0.148) (Fig. 4c). Furthermore, the result of LEfSe analysis demonstrated that the CKD transferred mouse group showed increased Clostridiaceae, *Clostridium* and *Parabacteroides*, as well as decreased Ruminococcaceae and *Megamonas*, which was in accordance with that of the CKD patients (Fig. 4d). In conclusion, the FMT from CKD patients led to increased TMAO levels and the dysbiosis of gut microbiota in mice receiving this.

## Discussion

CKD has become an important public health problem, and our study provides new information about the TMAO levels and gut microbiome in Chinese CKD patients. Our data demonstrated significantly elevated TMAO levels in CKD patients compared to healthy individuals (30.33 ± 27.35 μmol/L and 2.08 ± 1.89 μmol/L, respectively). Missailidis *et al*. reported that the TMAO levels in CKD 3–4 and CKD 5 patients and controls in Sweden were 73.5 μM/L, 14.6 μM/L and 5.8 μM/L, respectively, and that the renal functions were a major determinant of the TMAO level^22^. A comparison of the data from two studies showed that the TMAO levels of the healthy group were similar. In contrast, our patients’ TMAO levels were between the CKD 3–4 and CKD 5 patient group data from the previous study. In a clinical study in the USA, people with adverse cardiovascular events expressed significantly higher TMAO levels than the control group (5.0 µM and 3.5 µM, respectively)^12^. Compared with our former study, the TMAO level in Chinese atherosclerosis patients was similar to the control group but was deceased in stroke patients (2.92 μmol/L, 2.68 μmol/L, and 1.91 μmol/L, respectively)^17^, illustrating that in our study the TMAO level of the healthy controls was relatively normal and comparable, whereas the TMAO level was significantly higher in the CKD patients than in patients with other diseases. According to the findings of Tang *et al*., elevated TMAO levels in animal models were related to corresponding increases in tubulointerstitial fibrosis and Smad3 phosphorylation, which is an important regulator of fibrosis^15^. These data suggested that an extremely high TMAO level might be an important risk factor in CKD patients. Moreover, renal function is a key contributor that affects the TMAO concentration. Our results confirmed that a lower GFR resulted in a higher TMAO level. An inverse association between the TMAO level and the GFR has been observed in several studies^12, 22–24^. The GFR represents the renal filtration function, which is closely related to the amount of toxic metabolite excretion. Interestingly, in the Framingham Heart Study, TMAO was a predicative marker of CKD at 8 years of follow-up that was not related to the baseline GFR^25^, suggesting that TMAO may be a surrogate marker for the GFR and have nephrotoxic properties.

In accordance with the alteration in TMAO, severe dysbiosis of the gut microbial community was observed in the CKD patients. Shifts in the microbial community structure may play a role in the aggravation and development of CKD. In addition to deducing the change in gene functions through bioinformatics analysis, our study confirmed that the mouse group that received the gut microbiota from the CKD patients had a significantly higher TMAO level and different composition of gut microbiota than did the comparative mouse group. This result provided direct evidence for the first time that the increased TMAO levels in CKD patients were partially promoted by gut microbiome dysbiosis; additionally, the results indicated an essential role for the gut microbiota in TMAO generation and metabolism in CKD patients as previously reported^15^. However, we transferred the faecal samples from CKD patients into the antibiotic-treated mice for only 7 days and the time interval between the FMT and TMAO measurement was only 3 days. In this short term experiment, we deduced that the alteration of the microbial community mainly affected TMAO metabolism rather than renal functions. Additionally, from the PICRUSt analysis, we concluded that changes occurred in metabolites from the microbial populations and that the dysbiosis of the microbial community increased the TMAO levels, including choline, betaine, L-carnitine and TMA metabolism. Among the 8 genes, we found K07271, K01004, and K00499, which were associated with choline metabolism. The K00130 and K00540 genes were associated with betaine and L-carnitine metabolism, respectively. These genes were all relatively decreased in the CKD patients, suggesting that the altered gut microbiota led to abnormal choline, betaine, and L-carnitine metabolism, resulting in the production of redundant TMA in the intestinal tract. The genes relatively enriched in the CKD patients, including K07811, K07821, and K03532, were related to the increased reduction reaction of TMAO and the decreased decomposition of TMA, resulting in an elevated TMA concentration in the intestinal tract. These results initially suggested that the gut microbiome played a role as a potential source of uremic toxins, including TMAO. Studies employing poorly orally absorbed antibiotics coupled with the dietary intake of isotope-labelled phosphatidylcholine directly demonstrated an obligatory role for gut microbes in TMAO generation in humans^12^.

In addition to the causative role in TMAO production, the perturbed gut microbiota in CKD patients provided more information on the probable effect on host physiology. The healthy individuals in our study harboured more diverse and shared more similar microbial communities than did the CKD patients. A stable and diverse microbiota is commonly believed to be essential for host physiological processes and mucosal immune functions^26^. According to our results, many commensal or beneficial microbes, such as *Prevotella, Roseburia, Ruminococcaceae* and *Coprococcus*, were depleted in the patient group. These microbes generally exist in healthy populations belonging to the phylofunctional core of the intestinal microbiota^27^. These beneficial bacteria produce short chain fatty acids (SCFAs), especially butyrate acid, which has multiple critical roles in the maintenance of human health, including producing intestinal epithelial nutrition and energy components^28^, maintaining intestinal barrier functions^29^, reducing the severity of inflammation^30^, and enhancing colon motility functions^31^. Similarly, ESRD patients in the USA exhibited a reduction of bacterial families capable of producing SCFAs, which might contribute to uremic toxicity, systemic inflammation and adverse consequences in patients with CKD^32^. In contrast, CKD patients had a greater abundance of Proteobacteria, Enterobacteriaceae, Corynebacteriaceae, and *Enterococcus*, which are mainly opportunistic pathogens. The enrichment of Proteobacteria indicated an imbalanced and unstable microbial community structure of the host^33^. Enterobacteriaceae are among the most commonly overgrown symbionts in many conditions involving inflammation and present as the major harmful members in the human symbiotic microbial community. Inflammation-driven blooms of Enterobacteriaceae are associated with various diseases and potentially contribute to the pathogenesis of disease development^34^. Corynebacteriaceae belongs to non-pathogenic bacteria that can cause exceptional cases of opportunistic infections in immunocompromised humans^35^. Under this condition, these bacteria become colitogenic microbes that can trigger inflammatory responses. Similarly, Vaziri *et al*. found an increased abundance of Enterobacteriaceae, particularly OTUs containing certain clusters of *E. coli* sequences, in end-stage renal disease (ESRD) patients and a decreased abundance of the Prevotellaceae family in uremic animals^10^. Interestingly, no significant differences in the microbial community structure were observed between the GFR groups. By analyzing the raw microbiome data of CKD patients, the low GFR group harbored reduced *Streptococcus*, Streptococcaceae, and Lactobacillales, compared to the high GFR group. All of them are lactic acid bacteria (LAB) and traditionally considered to be beneficial microorganisms for human beings^36^. Besides, the low GFR group showed increased *Adlercreutzia*, which was positively correlated to the TMAO level (Spearman analysis, P = 0.002, R = 0.528), but the abundance of *Adlercreutzia* was relatively low (<0.00005). These results indicated that the difference of microbial composition between the low GFR group and the high GFR group may be involved in the TMAO separation between the two groups. However, the major contribution of this difference should be from the renal functions.

Moreover, contributory factors that could cause microbial dysbiosis in CKD patients were supported by evidence of clinical manifestations, impaired renal functions and dietary restrictions. According to a study in Belgium, 69 factors correlated significantly with microbiome community variations^37^, including red blood cell counts, haemoglobin, GFR and HDL cholesterol, which were in line with our discoveries. Impaired renal functions, such as protein assimilation, lead to the proliferation of proteolytic bacteria^38^, and increased protein fermentation can produce potentially toxic metabolites. Additionally, most of the CKD patients had dietary restrictions in an attempt to prevent hyperkalaemia, and Poesen *et al*. verified that dietary restrictions contributed to altered gut microbial metabolism in CKD patients to a large extent^39^. Although a direct mechanistic link between dietary choline or TMAO and the progressive impairment of renal functions has been verified in animal studies^15^, nutritional intervention studies investigating potential roles of choline or TMAO are rather limited in humans. Interestingly, *Bifidobacterium* has been reported to reduce the serum indoxyl sulfate level by correcting the microbial community in haemodialysis patients^40^, thereby modestly improving renal functions^41^.

The present study suggests that CKD significantly modifies the composition of the gut microbial community in humans, which also leads to the concentration and decreased excretion of TMAO. Animal experiments and the PICRUSt analysis further confirmed the essential role of the gut microbiota in TMAO generation and metabolism. Above all, we suggest that CKD patients have increased plasma TMAO levels due to contributions from both impaired renal functions and dysbiosis of the gut microbiota. Based on these findings, further research should be performed to identify the roles played by the gut microbiota in different CKD stages in the progression and aggravation of this disease. Significant microbiome differences with altered TMAO levels resulting in predicted perturbations of functional pathways could suggest a metabolite-specific targeted treatment.

## Methods

### Research participants and sample collection

We recruited chronic kidney disease patients in this study. All patients were consecutively recruited from the Department of Nephrology of Nanfang Hospital (a teaching hospital affiliated with Southern Medical University in southern China) during 2014. The following inclusion criteria were used: proven impaired kidney structure or renal function and a measured GFR < 60 mL/min/1.73 m^2^ for a minimum of 3 months. Patients with acute intercurrent illnesses and infections and those who used probiotics or antibiotics within 1 month before admission were excluded. The patient group (n = 32) consisted of thirty-two stable patients (16 men and 16 women, aged 53.34 ± 14.47 years) with abnormal renal functions for a minimum of 3 months. The underlying causes of CKD in this study population included nephrosclerosis in 10 patients, diabetic nephropathy in 5 patients, glomerulonephritis in 4 patients and polycystic kidney disease in 1 patient. The controls (n = 32, 16 men and 16 women, aged 55.03 ± 10.38 years) consisted of age- and gender- matched individuals from the health examination centre of Nanfang Hospital. The only exclusion criterion for the selection of the healthy controls was abnormal renal functions.

In our study, the CKD group were mainly constitutive of CKD4 and CKD5 patients. According to the new KDIGO (Kidney Disease: Improving Global Outcomes) classification, CKD stages are determined at referral and correspond to stages 4 (GFR, 15- < 30 mL/min/1.73 m^2^), and 5 (GFR < 15 mL/min/1.73 m^2^)^42^.

In this study, the GFR of each healthy participant was beyond 90 ml/min/1.73 m^2^. According to the Initiating Dialysis Early and Late (IDEAL) study^43^, we further divided the CKD patients into 2 subgroups. The high GFR group was determined as those patients whose GFR was higher than or equal to 7 ml/min/1.73 m^2^ and the low GFR group was determined lower than 7 ml/min/1.73 m^2^. Specifically, the low GFR group contained 16 CKD5 patients, and the high GFR group contained 15 CKD5 patients and 1 CKD4 patient.

For all participants, we collected fasting EDTA-plasma samples, fresh faecal samples and associated clinical data. Plasma aliquots were collected before dialysis treatment, centrifuged, and then immediately frozen at −40 °C. The faecal sample aliquots were frozen at −40 °C immediately after collection. The Ethics Committee of Southern Medical University approved the study protocol, and informed written consent was obtained from all participants. We confirmed that all methods were performed in accordance with the relevant guidelines and regulations.

### Faecal DNA extraction

DNA was extracted from the microbial flora contained in the stool samples using the PowerSoil DNA Extraction Kit (Shenzhen Bioeasy Biotechnologies. Co., Ltd) following the manufacturer’s specifications^44^.

### Polymerase chain reaction amplification of bacterial 16S rRNA genes

All 64 individually processed human faecal gDNA extractions were PCR amplified. The V4F (GTGTGCCAGCMGCCGCGGTAA) and V4R (CCGGACTACHVGGGTWTCTAAT) primers were used to amplify the 16S rRNA gene V4 variable region from the bacteria. The polymerase chain reaction cycle conditions were described previously^45^. Amplifications were performed using a step cycling protocol consisting of 94 °C for 2 minutes, 30 cycles of 94 °C for 30 seconds, 54 °C for 30 seconds, and 72 °C for 45 seconds. A condition of 72 °C for 5 minutes was used for the final elongation. Each 25-μL PCR reaction contained 0.5 μL of template DNA, 2.0 μL of dNTP Mix (2.5 mmol/L; TaKaRa), 2.5 μL of TaKaRa 10× Ex Taq buffer (Mg2+ free), 1.5 μL of Mg^2+^ (25 mmol/L), 0.25 μL of TaKaRa Ex Taq DNA polymerase (2.5 units), 0.5 μL of 10 μmol/L bar code primer 514 F, 0.5 μL of 10 μmol/L primer 805R, and 17.25 μL of double-distilled water.

### Sequencing and microbiome data analysis

According to the manufacturer’s protocol, all polymerase chain reaction amplicons were mixed together and sent to the Beijing Genomic Institute for sequencing using Illumina Miseq (PE 150). The raw sequences were preprocessed based on the BIPES protocol^45^. The sequences were analysed using QIIME version 1.8.0^46^. All samples were normalized for the subsequent analysis. The sequences were stored in the Sequence Read Archive database under accession numbers ERS725509 to ERS725741.

Sequences with a distance-based similarity of 97% or greater were grouped into operational taxonomic units (OTUs) using the Usearch algorithm^47^. According to the sequence frequency, we determined the representative sequences for each OTU, which were aligned using the PyNAST algorithms^48^. The phylogenetic relationships were determined based on a representative sequence alignment using Fast-Tree^49^. The taxonomic assignments for each representative sequence were measured, and the above information was combined to construct a BIOM file^50^ using the command pick_de_novo_otus.py –i clean.fasta –o output_dir. We performed principal coordinate analysis (PCoA) using the command beta_diversity_through_plots.py –i otu.biom –o output_dir.

To determine the significantly differential taxa between the CKD patients and the healthy individuals, we applied linear discriminant analysis effect size (LEfSe) to compare samples between these 2 groups^51^. The threshold of the linear discriminant was set to 3. LEfSe is an algorithm for high-dimensional biomarker discovery that also identifies genomic features that characterise differences between two or more biological conditions. LEfSe allows researchers to identify differentially abundant features that are also in accord with biologically meaningful categories by emphasizing statistical significance, biological consistency and effect relevance. For each of the differential features detected by LEfSe, we calculated a linear discriminant analysis value, which represented the differences of the feature between the tested groups.

### Quantification of the plasma TMAO concentrations

Plasma aliquots were stored at −40 °C prior to analysis. The plasma TMAO concentrations were quantified using stable isotope dilution liquid chromatography tandem mass spectrometry (6460 Series Triple Quadrupole LC/MS; Agilent) as previously described^52^.

The molecular weight of TMAO is 75.22 g/mol. The extracted plasma aliquots were spiked with internal standards comprised of d9-TMAO in methanol. The samples were mechanically vortexed for 1 minute and centrifuged at 15,000 × g for 25 minutes at 4 °C. The analytes were separated on a phenomenex Luna Silica column (4.6 mm × 250 mm, 5 μm particle size) at room temperature. The mobile phase consisted of 80% solvent A (0.1% formic acid in water) and 20% solvent B (methanol) at a flow rate of 0.5 mL/min. The MS/MS analyses for TMAO and d9-TMAO were conducted in the positive multiple reaction monitoring mass spectrometry mode at m/z 76 → 58 and m/z 85 → 66, respectively. To calculate the TMAO concentration, various concentrations of a TMAO standard were added to control plasma to prepare calibration curves. The data were collected and analysed using the chemistry station provided by Agilent Technology.

### Animal experiments

The animal experiment protocols were reviewed and approved by the Ethical Committee of Southern Medical University (SMU, Guangzhou, China). All procedures were performed in accordance with the relevant guidelines and regulations. Male C57BL/6 mice were maintained at the Experimental Animal Research Centre at Southern Medical University. The mice were given the same autoclaved standard chow and autoclaved water ad libitum. The animal room was controlled at 23 °C and 40% humidity with a 12-h light/12-h dark cycle. The general health status of the mice, including their physical appearance, was checked every day. All mice in this study had comparable normal health statuses before the experiment.

At the start of the experiment, all mice were 4 weeks old. All 25 mice were treated with a mixture of broad spectrum, poorly absorbed antibiotics, including vancomycin (100 mg/Kg), neomycin sulfate (200 mg/Kg), metronidazole (200 mg/Kg), and ampicillin (200 mg/Kg) for 1 week, in accordance with a previous study^53^. After this procedure, the mice were randomized into two groups with 12 and 13 mice in each group, respectively. Both groups were treated with faecal microbiota transplantation (FMT) and transplanted with the faeces of the CKD patients or the healthy controls. These two groups of mice were separately bred in different isolators to prevent normalization of the gut microbiota.

Faecal samples from randomly chosen CKD patients (n = 5, male) and healthy controls (n = 5, male) were used. Each faecal sample (0.1 g) was suspended in 1.5 mL of reduced sterile phosphate-buffered saline, and pools were made from equal volumes of the donor suspensions. Adult (5 weeks old) male antibiotic-treated C57BL/6 mice were administered pooled samples derived from either the CKD patients or the healthy controls for 1 week. After centrifugation of the suspensions at 2000 rpm for 10 minutes, the supernatants were collected, and 200 μL was administered to each mouse by gastric gavage.

On day 3 post-FMT, plasma samples were obtained from the mice and immediately stored at −40 °C. These samples were analysed by stable isotope dilution liquid chromatography tandem mass spectrometry to determine the TMAO levels in the mice. The cecum contents were collected when the mice were sacrificed and immediately stored at −40 °C.

### Bioinformatics analysis and statistical analyses

Here, we used PICRUSt (Phylogenetic Investigation of Communities by Reconstruction of Unobserved States) to gain insights into the possible functional pathways that differed between groups^54^. PICRUSt uses evolutionary modelling to predict metagenomes from 16S rDNA-derived bacterial data and a reference genome database.

Statistical analysis of the clinical data was implemented using SPSS 20.0. We used the mean (standard deviation) to express measurement data that obeyed a normal distribution, the median (interquartile range) to express measurement data that obeyed a skewed distribution, and a percentage to express enumeration data. The Normality test was used to determine the distribution of the data, and the Mann-Whitney U test was used to determine significance between different groups. The Wilcoxon rank sum test was used to determine significance in α-diversity. The results of Mann-Whitney U test was two-tailed. P < 0.05 was considered significant in the compared groups.

## Electronic supplementary material

Supplementary Information
