# Supplementary material for: Impaired renal function and dysbiosis of gut microbiota contribute to increased trimethylamine-N-oxide in chronic kidney disease patients
Source: Sci Rep. 2017 May 3;7:1445. doi: 10.1038/s41598-017-01387-y (PMC5431124; doi:10.1038/s41598-017-01387-y)
Supplement: Supplementary file 1 — Supplementary Information [file 41598_2017_1387_MOESM1_ESM.pdf]

# Impaired renal function and dysbiosis of gut microbiota contribute to increased trimethylamine-N-oxide in chronic kidney disease patients

Short title: Increased TMAO and gut microbiome dysbiosis in CKD patients

Kai-Yu Xu[1#], Geng-Hong Xia[1#], Jun-Qi Lu[2,3#], Mu-Xuan Chen[3], Xin Zhen[4], Shan Wang[3], Chao You[1], Jing Nie[4], Hong-Wei Zhou[3]*, Jia Yin[1]*

[1] Department of Neurology, NanFang Hospital, Southern Medical University, Guangzhou, China

[2] Department of Environmental Health, School of Public Health, Southern Medical University, Guangzhou, China

[3] State Key Laboratory of Organ Failure Research, Division of Laboratory Medicine, ZhuJiang Hospital, Southern Medical University, Guangzhou, China

[4] Department of Nephrology, NanFang Hospital, Southern Medical University, Guangzhou, China

[#] Kai-Yu Xu, Geng-Hong Xia and Jun-Qi Lu contributed to this work and share the first authorship.

* Jia Yin and Hong-Wei Zhou share the last authorship.

Correspondence to：

Dr. Jia Yin, Department of Neurology, NanFang Hospital, Southern Medical University, Guangzhou, China, 510515.

Fax: +86 020 62787664

Tel: +86 020 61641965

Email: jiajiayin@139.com

Prof. Hong-Wei Zhou, State Key Laboratory of Organ Failure Research, Division of Laboratory Medicine, ZhuJiang Hospital, Southern Medical University, Guangzhou, China, 510515.

Fax: +86 20 61648327

Tel: +86 20 61648327

Email: zhou_hong_wei@qq.com

**Supplement Figure 1**

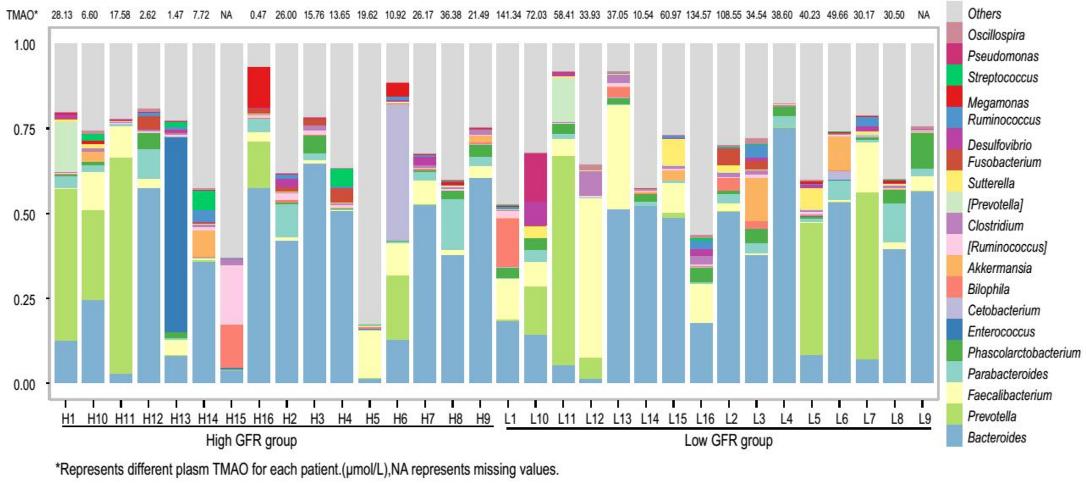


**The raw microbiome data and TMAO concentrations of CKD patients.** The data represent the raw gut bacterial profiles of CKD patients, including the high GFR group and the low GFR group. Average relative abundances of the predominant bacterial taxa at the genus level and the TMAO concentrations of each CKD patient were showed.
